# Race-related host and microbe transcriptomic signatures in triple-negative breast cancer

**DOI:** 10.1038/s41523-025-00806-y

**Published:** 2025-08-08

**Authors:** Roshan Kumar, Susan Duyar-Ayerdi, Aishwarya Sundaresan, Vinodh Srinivasasainagendra, Chandra Sekhar Pedamallu, Michael Behring, Darshan Shimoga Chandrashekar, Isam-Eldin Eltoum, Sooryanarayana Varambally, Hemant K. Tiwari, Sadeep Shrestha, Paul L. Auer, Lubna N. Chaudhary, John R. Kirby, Clayton Yates, Upender Manne, Akinyemi I. Ojesina

**Affiliations:** 1 Department of Obstetrics and Gynecology, Medical College of Wisconsin, Milwaukee, WI USA; 2 Medical College of Wisconsin Cancer Center, Milwaukee, WI USA; 3 Post-Graduate Department of Zoology, Magadh University, Bodh-Gaya, Bihar, India; 4 Department of Biostatistics, University of Alabama at Birmingham, Birmingham, AL USA; 5 Excelra, Boston, MA USA; 6 Department of Pathology, University of Alabama at Birmingham, Birmingham, AL USA; 7 Department of Epidemiology, University of Alabama at Birmingham, Birmingham, AL USA; 8 Division of Biostatistics, Data Science Institute, Medical College of Wisconsin, Milwaukee, WI USA; 9 Department of Medicine, Division of Hematology and Oncology, Medical College of Wisconsin, Milwaukee, WI USA; 10 Department of Microbiology and Immunology, Medical College of Wisconsin, Milwaukee, WI USA; 11 Department of Pathology, Johns Hopkins School of Medicine, Baltimore, MD USA; 12 Department of Oncology, Sidney Kimmel Comprehensive Cancer Center, Johns Hopkins University School of Medicine, Baltimore, MD USA

**Keywords:** Cancer genomics, Microbiology, Breast cancer

## Abstract

Triple-negative breast cancer (TNBC) shows racial disparities, with higher incidence in women of African ancestry (AA) compared to European ancestry (EA). Meta-transcriptomic analysis of TNBC tumor tissues from AA (n = 17) and EA (n = 19) subjects revealed distinct microbial landscapes. Hierarchical clustering based on microbial transcripts separated samples into two groups predominantly defined by racial ancestry. Bacterial genera including *Hafnia* and *Cedecea* were more abundant in AA tumors, while *Erwinia* was higher in EA tumors. Cellular composition analysis by xCell revealed differences in immune cell populations, with AA tumors having higher Th1 cell abundance and EA tumors containing higher macrophage M2 cell abundance. Nonetheless, AA women with high M2 abundance experienced poorer disease-free survival (DFS) than EA women. Integrative analyses revealed that high expression of human *SPDYE2B* gene was associated with *Hafnia* abundance and decreased DFS, highlighting complex host-microbe interactions in TNBC patients.

## Introduction

Breast cancer represents a preeminent health burden, leading to approximately 15% of cancer-related deaths in women worldwide. In the United States about 13 of every 100 women are expected to develop breast cancer during their lifetime^1^. Triple-negative breast cancer (TNBC), a highly aggressive form of breast cancer is characterized by the absence of estrogen receptor (ER), progesterone receptor (PgR), and human epidermal growth factor receptor-2 (HER2)^2^ as determined by immunohistochemistry^3^. Patients diagnosed with TNBC face an increased risk of distant metastasis and mortality within the first 5 years of diagnosis^4^.

Various factors, both genetic and non-genetic, are associated with an elevated risk for TNBC^5–8^. TNBCs exhibit racial disparities, with higher incidence and mortality rates in women of African ancestry (AA) compared to those of European ancestry (EA)^9–11^. AA women tend to be diagnosed at younger ages and more advanced disease stages^12,13^. Although socioeconomic differences contribute to these disparities^14,15^, biological factors are also involved in TNBC risk and outcomes for AA patients^8^. Well-documented pathogenic variants in the *BRCA1* and *BRCA2* genes partially determine breast cancer susceptibility, and many new therapies target known genetic drivers^16^. Our previous studies show gene expression differences between AAs and EAs^17,18^. However, the contribution of environmental factors remains poorly understood. Recent studies reveal diverse microbial communities in normal and malignant breast tissue^19–29^, with tumor type-specific bacteria present in both cancer and immune cells^29^. In the present study, we aimed to provide insights into the potential interplay between the tumor microbes and host genes in AA and EA patients and their associations with prognostic outcomes.

## Results

### Patient cohort

We investigated 17 AAs and 19 EA samples, a subset of tumors defined by genetic ancestry models in larger, recently described RNASeq studies^17,18,30^ of women with TNBCs, diagnosed at the University of Alabama at Birmingham (UAB) between 2001 and 2012 (Table 1). The samples used were approved by the Institutional Review Board at UAB (IRB number: 060911009). When available, clinical information such as age, gender, date of diagnosis, and pathological stage (according to the American Joint Committee on Cancer [AJCC] staging system) of the tumor, determined after surgical resection, was obtained from the patients’ medical records. Metastatic staging was not performed unless clinically indicated. Therefore, all patients were considered by their treating surgeons to have localized, non-metastatic cancer.

**Table 1:** Race and clinical attributes of the selected patients

	Level	AA	CA	*p* value
Sample Size		17	19	
Stage (%)	II	10 (58.8)	18 (94.7)	0.006
	III	7 (41.2)	0 (0.0)	
	IV	0 (0.0)	1 (5.3)	
Grade (%)	II/III	2 (11.8)	1 (5.3)	0.92
	III/III	15 (88.2)	18 (94.7)	
Neoadjuvant Chemotherapy (%)	No	11 (64.7)	16 (88.9)	0.194
	Yes	6 (35.3)	2 (11.1)	
Adjuvant Chemotherapy (%)	No	3 (23.1)	1 (7.1)	0.534
	Yes	10 (76.9)	13 (92.9)	
Radiation (%)	No	6 (40.0)	6 (37.5)	1
	Yes	9 (60.0)	10 (62.5)	

**Fig. 1:**
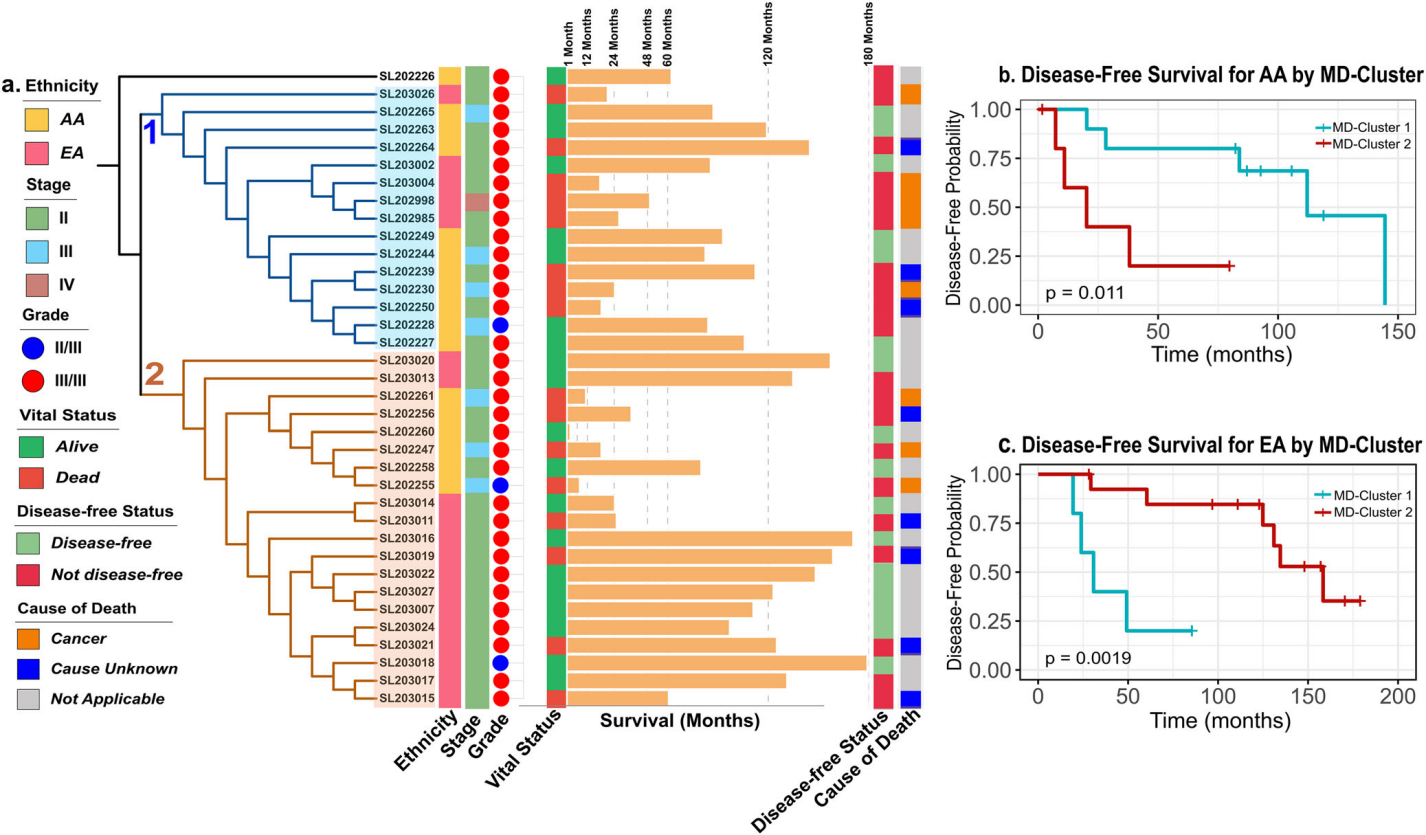
Clinicopathological features of microbial transcript-based tumor MD-Clusters. **a** Hierarchical clustering separated tumors into two major MD-Clusters by microbial transcript profiles. MD-Cluster 1 predominantly contained AA samples (nAA = 10), while MD-Cluster 2 was enriched for EA samples (nEA = 14). Within each MD-Cluster, sub-clusters further segregated by race. Metadata associated with each sample, including stage, grade, vital status, disease-free status (Disease-free: alive without disease or unknown status; Not disease-free: dead or alive with confirmed disease), and cause of death, is displayed. **b**, **c** Kaplan–Meier analysis of disease-free survival (DFS) for AA and EA, respectively, based on MD-Clusters.

The survival time ranged between 1.7 months after diagnosis to over 14.8 years (Fig. 1a). By the end of the follow-up period (median = 84.7 months), 16 of the 36 patients had died due to cancer. Of the 20 patients alive, 14 were considered disease-free (Fig. 1a). The clinical stage at diagnosis spanned between stages II to IV, with most being stage II, followed by stage III (Fig. 1a). There were significantly more EA patients with stage II than AA patients (*p* = 0.006) (Table 1). Concordant with what was expected among TNBCs, most patients had grade III tumors regardless of race. Patients who received radiation in addition to chemotherapy (neoadjuvant and/or adjuvant) had better DFS than those without radiation treatment, but there was no statistically significant difference in DFS between AA and EA patients (Supplementary Fig. 1).

### Microbial abundance and cluster analysis

RNA sequencing of macro-dissected breast tumor samples was performed using standard methods^17,18,30^. The identity and abundance of microbial transcripts in tumor samples were determined using the PathSeq computational subtraction pipeline^31,32^. Unsupervised hierarchical clustering of relative microbial abundance data using Spearman rank correlation revealed two major microbe-derived (MD) clusters, MD-Cluster 1 and MD-Cluster 2 (Fig. 1a). Interestingly, these MD-Clusters exhibited race-related separation (Fisher’s exact p = 0.044). MD-Cluster I was predominantly comprised of AA patients (ntotal = 15, nAA = 10); MD-Cluster II was dominated by EA patients (ntotal = 20, nEA = 14). Within each MD-Cluster, samples formed sub-clusters by racial group. In the AA cohort, patients in MD-Cluster 1 had better DFS than those in MD-Cluster 2 (Fig. 1b), whereas, in the EA cohort, patients in MD-Cluster 2 had better DFS than those in MD-Cluster 1 (Fig. 1c).

**Fig. 2:**
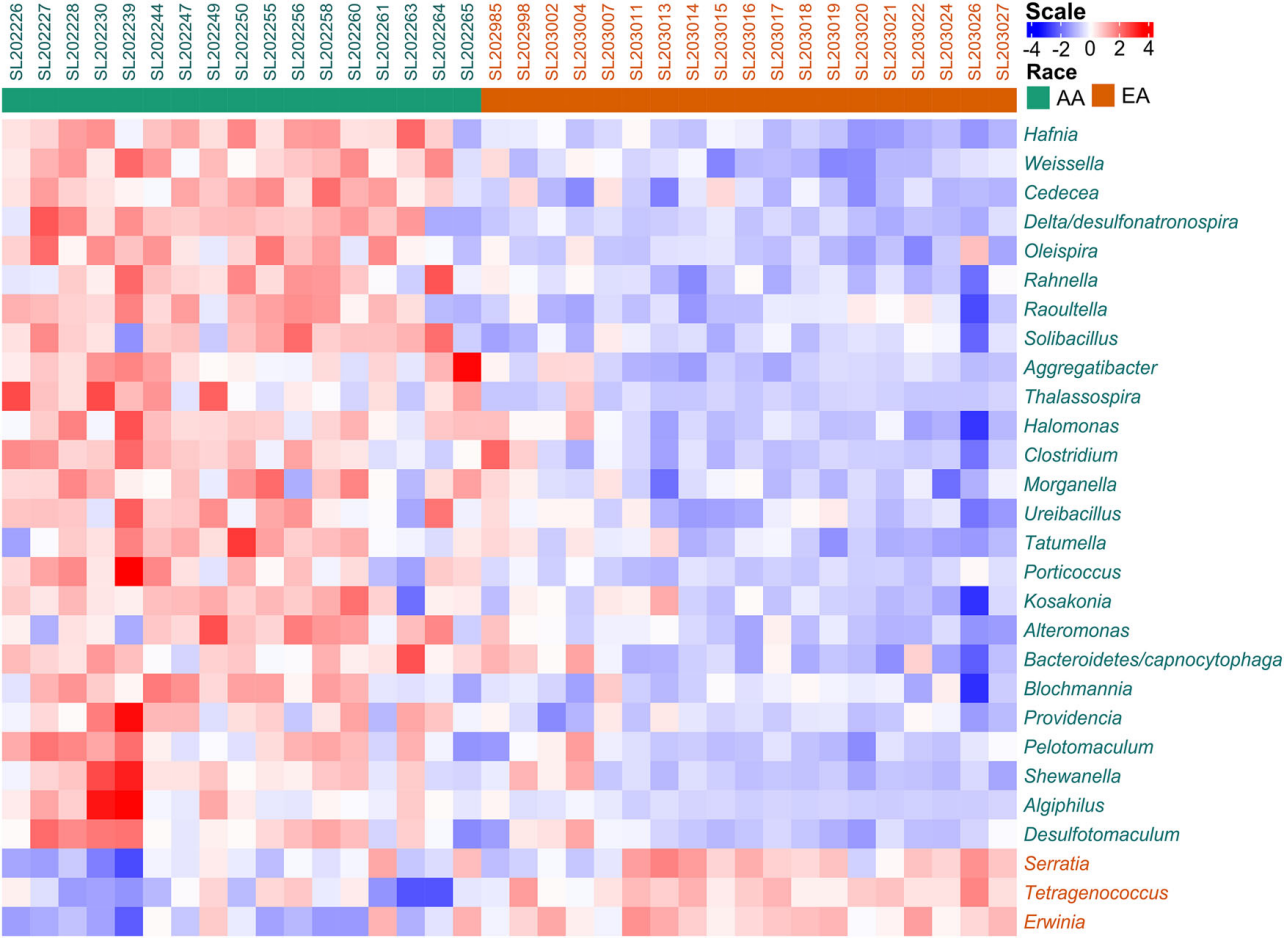
Differential abundance of tumor microbiome in tnbc patients by race. Comparative marker selection analysis was performed using t-tests (FDR < 0.05) to identify taxa with differential abundance between AA and EA patients.

The most abundant genera in both AA and EA tumors were similar: *Bacillus, Escherichia, Serratia, Shigella*, and *Cronobacter* (Supplementary Data 1). However, there were significant differences in relative microbial abundance in tumors across the two racial groups (Fig. 2). *Hafnia, Weisella, Cedecea, Delta/Desulfonatronospira, Olespira, Rahnella, Raoultella, Solibacillus, Kosakonia, Aggregatibacter, Thalassospira*, *Halomonas*, and *Clostridium* species exhibited significantly higher abundance (FDR ≤ 0.05) in the AA population. In contrast, *Erwinia*, *Tetragenococcus*, and *Serratia* showed significantly higher abundance (FDR ≤ 0.05) in the EA patients (Fig. 2). Additionally, the differential abundance of microbes based on MD-Clusters (Supplementary Fig. 2) identified *Serratia* was notably abundant in MD-Cluster 2, which was predominantly comprised of EA patients. Interrogation of the Microbe Atlas Project database (https://microbeatlas.org/) revealed that the above-listed bacterial genera have been previously detected in human samples.

### Differential expression of genes based on race and MD-Cluster

**Fig. 3:**
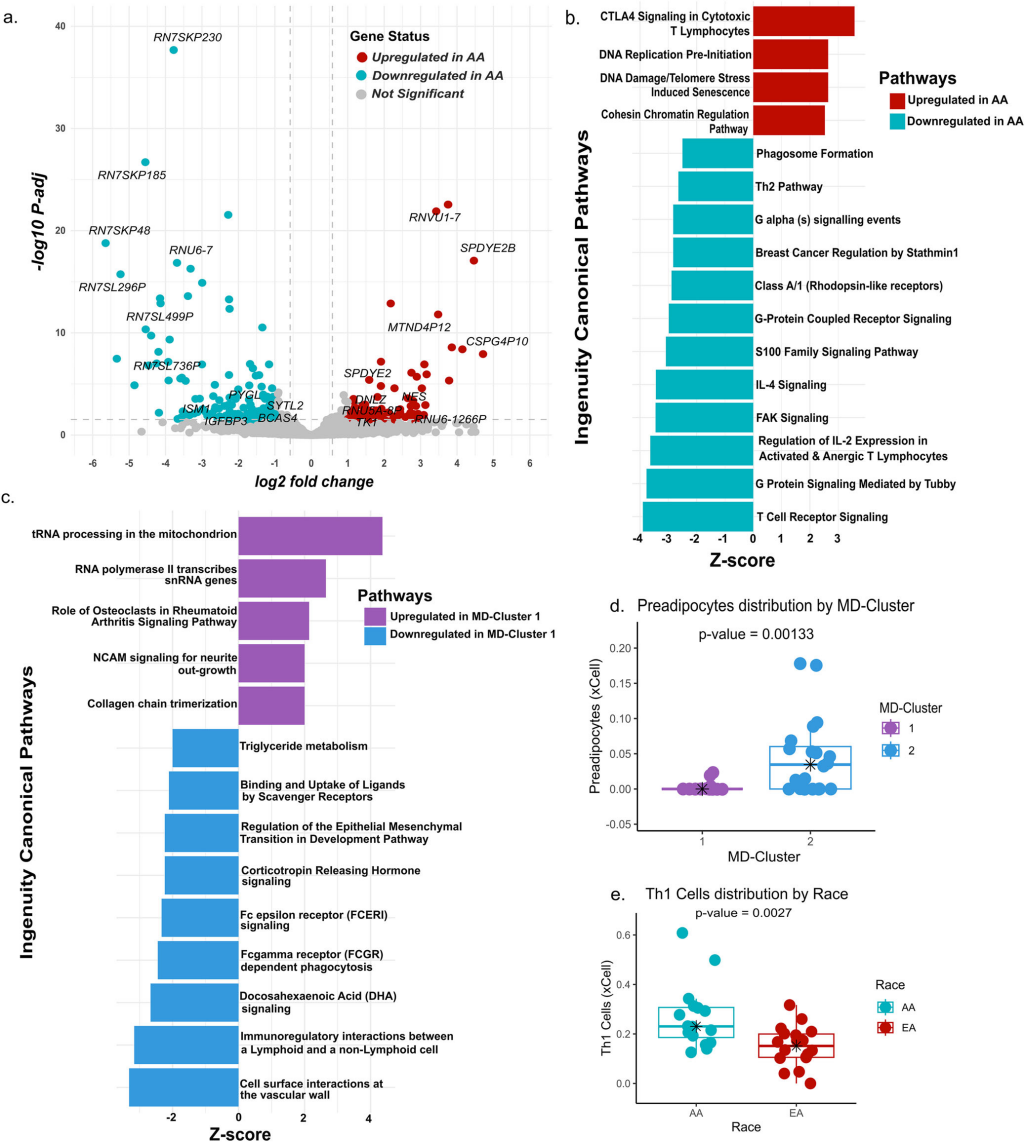
Differentially expressed genes and related pathways in TNBC patients. (**a**) Volcano plot showing the log2 fold change and adjusted p-values for all differentially expressed genes between AA and EA cohorts ( | log2FoldChange | ≥ 1, padj < 0.05). **b**, **c** Bar charts depicting canonical pathways predicted to be differentially activated in AA (red) versus EA (cyan), and MD-Cluster 1 (violet) vs MD-Cluster 2 (bright blue), respectively. Pathways were identified based on a Z-score cutoff of ±2 and a p-value of <0.05. Detailed lists of genes and pathways are available in Supplementary Data 2, 3. **d**, **e** Box plots of the xCell scores for preadipocytes by MD-Cluster and Th1 cells by race, respectively.

Differential expression analysis of human RNASeq data by race revealed 40 differentially upregulated (L_2_FC ≥ 1 and FDR ≤ 0.05), and 129 differentially downregulated genes in AA patients compared with EA patients (L_2_FC ≤ −1 and FDR ≤ 0.05), including protein-coding and non-coding genes (Fig. 3a, Supplementary Data 2). Of these, the top 5 differentially upregulated (by L_2_FC) coding genes in AAs were *SPDYE2B, HIST1H4F, NES, SPDYE2*, and *HIST1H3I*, while the top 5 non-coding upregulated genes in AAs were *CSPG4P10*, *RNU6-1266P*, *MTND4P12*, *TCEB3CL2*, and *CSPG4P8*. In contrast, the top 5 downregulated coding genes in AAs were *ISM1, IGFBP3, PYGL, BCAS4*, and *SYTL2*, while the top 5 non-coding downregulated genes in AAs were *RN7SKP48*, *MTRNR2L10*, *MTRNR2L11*, *RP11-777B9.5*, and *RN7SL499P*.

Ingenuity Pathway Analysis (IPA; Qiagen) revealed several significant canonical pathways (*p* ≤ 0.05) with differential expression between AA and EA patients. DNA replication pre-initiation and DNA damage/Telomere stress-induced senescence were upregulated in AAs compared with EAs (Fig. 3b), with higher expression of histone H2A and H2B family genes, such as *H2AC14, H2AC7, H2BC12, H2BC14, H2BC15*, and *H2BC9* (Supplementary Data 3). In contrast, the stathmin1 pathway, the Th2 pathway, the S100 family signaling pathway, and IL-4 signaling were downregulated in AAs (Fig. 3b). A list of genes involved in these pathways is provided in Supplementary Data 3.

Differential gene expression analysis of the MD-Clusters revealed 31 upregulated (L2FC ≥ 1 and FDR ≤ 0.05) and 64 downregulated genes (L2FC ≤−1 and FDR ≤ 0.05) (Supplementary Fig. 3) in MD-Cluster 1. The top 5 upregulated coding genes were *PRRG3, MT1H, RNY5, LRRD1* and *WDR93* while the top 5 non-coding genes were *SNORD21*, *MT-TF*, *CTD-2651B20.7*, *SNORD55* and *SNORD23*. The top 5 downregulated coding genes were *SPDEF*, *CXCL17*, *CHRDL1*, *IGF1* and *FCRL5*, while the top 5 downregulated non-coding genes were *CTD-2545G14.7*, *TRAJ33*, *TRAJ39*, *TRAJ30* and *SSXP10* (Supplementary Data 4).

IPA revealed upregulation of genes engaged in tRNA processing in mitochondria and RNA polymerase II transcription of snRNA genes in MD-Cluster 1 (Fig. 3c). Conversely, triglyceride metabolism and docosahexaenoic acid (DHA) pathways were downregulated in MD-Cluster 1. A list of the genes involved in these pathways is provided in Supplementary Data 5.

Next, we investigated the cell type enrichment in the tumors using xCell^33^. The analysis revealed significant enrichment (*p*-value ≤ 0.05) of preadipocytes in MD-Cluster 2 (Fig. 3d). In addition, Th1 cells, megakaryocyte-erythroid progenitor (MEP) cells, and pro B-cells in AA patients and M2 macrophages in EA patients (Fig. 3e, Supplementary Fig. 4A). Interestingly, a linear relationship was observed between M1 and M2 abundance in AA but not in EA women (Supplementary Fig. 4B), and survival analysis with patient stratification by race and M2 abundance revealed that AA women with high M2 abundance had poorer DFS than other subgroups (Supplementary Fig. 4C).

### Integrated analysis of microbial and host gene abundance and their relationship to disease-free survival

**Fig. 4:**
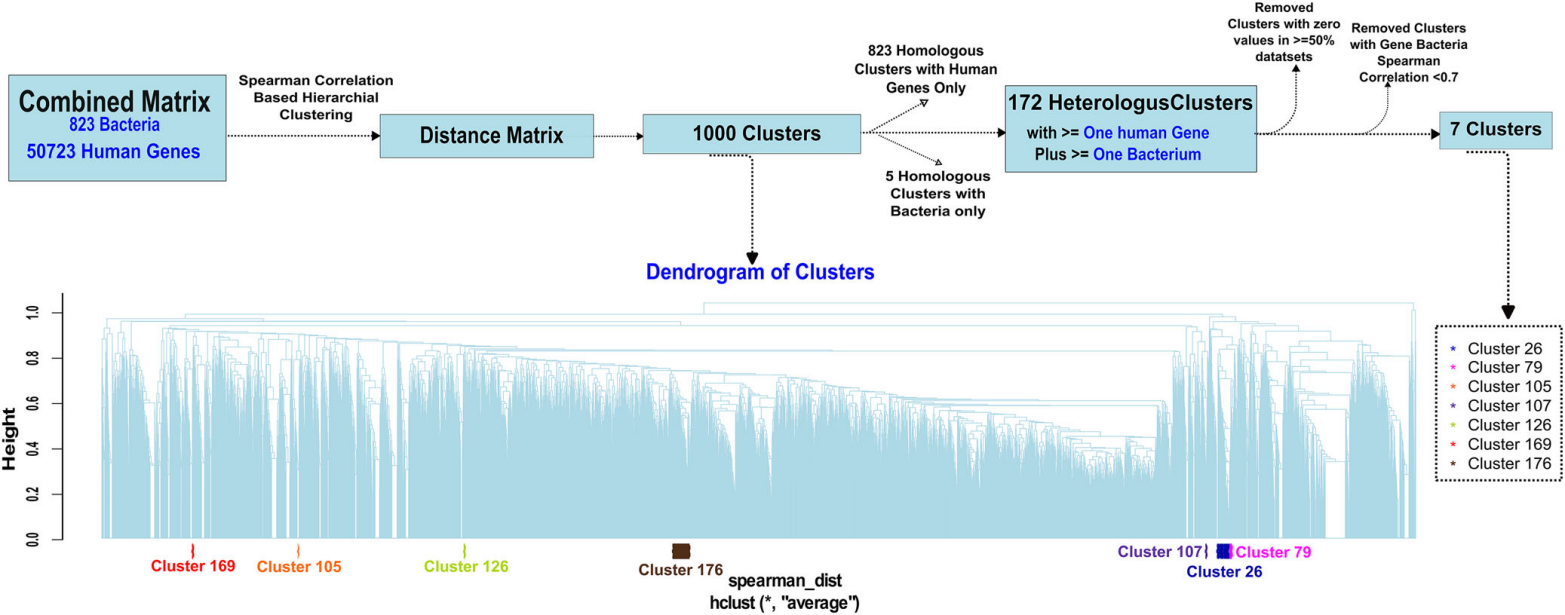
Hierarchical clustering-based relationships between host genes and bacteria. Host genes and microbial transcripts were hierarchically clustered based on co-abundance patterns, with homologous clusters containing either host genes only or bacteria only filtered out to retain high-confidence heterologous clusters containing both host genes and bacteria with a Spearman correlation >0.7.

To identify relationships between the microbiome and the host transcriptome, we combined microbial transcript abundance and host gene expression data into a single matrix. Hierarchical clustering using Spearman rank correlation was performed on the combined data matrix. Of the 1000 clusters generated, there were 828 homologous clusters (823 with host genes only, 5 with bacteria only) while 172 heterologous clusters contained at least one host gene and one bacterium (Fig. 4, Supplementary Data 6). There were 7 “high-confidence” heterologous clusters, which displayed strong positive correlative relationships (Spearman rank R > 0.7) between microbial abundance and host gene expression (Supplementary Data 7).

**Fig. 5:**
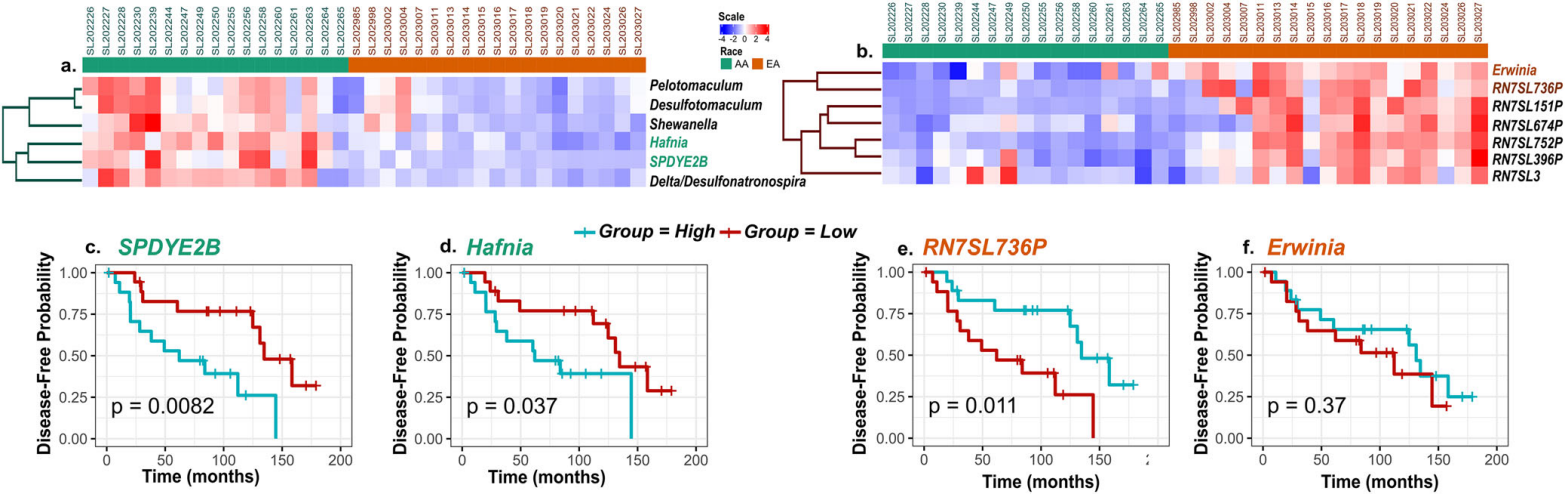
Correlative and survival relationships between host gene expression and microbial transcript abundance. **a** Sub-cluster from cluster 26 showing clustering of *SPDYE2B* gene with *Hafnia*, *Desulfotomaculum*, *Pelotomaculum*, *Shewanella*, and *Delta*/*Desulfonatronospira*. **b** Sub-cluster from cluster 176 showing clustering of *RN7SL736P* pseudogene with *Erwinia*. **c** Kaplan–Meier (KM) analysis of DFS by *SPDYE2B* gene expression. **d** KM analysis of DFS by *Hafnia* abundance. **e** KM analysis of DFS by *RN7SL736P* gene expression. **f** KM analysis of DFS by *Erwinia* abundance.

Cluster 26 revealed two notable relationships. In one sub-cluster, the host gene *SPDYE2B* closely clustered with *Hafnia* (R = 0.77), *Shewanella* (R = 0.74), *Delta/Desulfonatronospira* (R = 0.69), *Desulfotomaculum* (R = 0.66), and *Pelotomaculum* (R = 0.66). These features were more abundant in AA patients (Fig. 5a). Another sub-cluster of cluster 26 showed close clustering of the *SCARNA4* gene with *Shinella* (R = 0.79). Notably, *Erwinia* abundance was highly correlated in a sub-cluster of cluster 176 with levels of the *RN7SL736P* pseudogene (R = 0.756), which was more abundant in EA patients (Fig. 5b). Other highly correlated host-microbe associations (Supplementary Data 7) included *SNORA18* and *Halothiobacillus* in cluster 79 (R = 0.704), *BLOC1S1* and *Desulforudis* in cluster 105 (R = 0.732), *IGLVI-70* and *Delta/Arcobacter* in cluster 107 (R = 0.723), *KRT5* and *Heliobacterium* in cluster 126 (R = 0.73), *TMEM203* and *Idiomarina* in cluster 169 (R = 0.72), and *CCNYL1* and *Oceanobacillus* in cluster 174 (R = 0.72).

We evaluated the relationship(s) between these features (microbe(s)/gene(s)) and DFS (Fig. 5c–f, Supplementary Fig. 4). For each host gene or microbe, the cohort was stratified into high and low groups determined by the median values for each feature. High expression of the host gene *SPDYE2B* was associated with reduced DFS (*p* = 0.0082) (Fig. 5c). Further, high abundance of *Hafnia* (Fig. 5d) or *Shewanella* (Supplementary Fig. 4) were also associated with worse DFS (p = 0.037 and *p* = 0.012, respectively). However, although high *RN7SL736P* levels were associated with better DFS (*p* = 0.011), there was no association between its correlated microbe *Erwinia* and DFS (Fig. 5e, f).

In a Cox proportional hazards model adjusting for race, MD-Cluster, stage, and treatment type, only a high abundance of *SPDYE2B* and race remained associated with worse DFS (Supplementary Data 8).

## Discussion

Host genes affect the pathogenesis and survival outcomes of patients with TNBCs^5,6,34^. The variability in the tumor microenvironment (TME), is involved in shaping the transcriptomic landscape and driving intratumor heterogeneity across various malignancies^35,36^ and these fluctuations can alter the growth of microbiota within the TME^37^. Given this dynamic interplay, we investigated the relationships between the tumor microbiome, host transcriptome and outcomes in TNBC patients. For this analysis, we extracted RNA from formalin-fixed paraffin-embedded (FFPE) tissue samples and performed microbial analysis using PathSeq. The use of FFPE samples for microbiome research has gained increased traction in recent^34–36^. A comparative study between FFPE and fresh frozen samples demonstrate that 78.55% of operational taxonomic units remain consistent across preservation methods, with no significant differences observed in core bacterial composition and alpha diversity^37^. Other studies^34–36^, have established the feasibility and reliability of retrospective microbiome studies using archived clinical specimens- which represent the most widespread sample type available in cancer research.

Microbial transcript-based hierarchical clustering of TNBC patients (n = 36) revealed two distinct microbiome-derived clusters (MD-Clusters), each dominated by samples from different racial cohorts. We identified relatively higher abundance of *Hafnia* and *Shewanella* in AA tumors, and *Erwinia* and *Serratia* in EA tumors. The differential abundance of bacterial species by race suggests a role for these microbes in modulating tumor biology and influencing clinical outcomes in a race-dependent manner. Notably, *Hafnia* species are frequently isolated from clinical specimens and are often associated with extra-intestinal infections, particularly in immunocompromised individuals^38,39^. Although *Hafnia* is currently being marketed as a probiotic with anorexigenic properties^40^, *Hafnia* infections have also been associated with fatal outcomes^41^. *Hafnia* produces a cytolytic toxin active against Vero cells^42^ and has been shown to survive within HeLa cells^43^. These contrasting observations suggest the need for further studies on the pathogenic potential of this bacterial genus. Although our understanding of the factors that may potentially contribute to *Hafnia* pathogenesis within a host is limited, suggested mechanisms include biofilm formation, motility, and adherence to eukaryotic cells^44^. Similarly, *Shewanella*, though a rare pathogen in humans, has been associated with pulmonary and blood infections in cancer patients, indicating its relevance in the context of immunocompromised individuals and its capacity to affect TME^45,46^. The microbes significantly abundant in EA patients, such as *Serratia*, cause infections in cancer patients^47^. The substance known as Coley’s toxin, formed by the toxins produced by *Serratia marcescens* and *Streptococcus pyogenes*, induces tumor regression and has been successfully used to treat a variety of cancers, including sarcomas, carcinomas, lymphomas, myelomas, and melanomas^48^.

We observed race-associated differential expression in both protein-coding and non-coding RNAs (ncRNAs) genes. Two of the most differentially expressed coding genes in AA patients (*SPDYE2* and *SPDYE2B)* encode proteins that belong to the Speedy/Ringo family of non-canonical activators of the cyclin-dependent kinases, CDK1 and CDK2, which control cell cycle progression^49,50^. Overexpression of the prototypical family member RingoA/Spy1 has been observed in breast cancer tissue microarrays, and siRNA knockdown of RingoA/Spy1 results in decreased proliferation of the TNBC cell line MDA-MB-231^51,52^. Therefore, it is possible that high expression of these Speedy/Ringo genes may differentially modulate cell cycle regulation in AA TNBC patients.

Some of the top upregulated genes in the AA cohort included pseudogenes and ncRNAs, such as *CSPG4P10*, *RNU6-1266P*, *RP11-17J14.2*, and *MTND4P12*, suggesting potential regulatory roles for race-associated pseudogenes in TNBC pathogenesis. For example, the pseudogene *MTND4P12* functions as a competing endogenous RNA (ceRNA), regulating the expression of the well-established oncogene AURKB^53^. By sequestering microRNAs that would otherwise target and repress AURKB mRNA, *MTND4P12* may facilitate the aberrant expression of this kinase, leading to tumor growth and metastasis. Interestingly, *MTND4P12* expression is negatively correlated with overall survival in cutaneous melanoma patients^53^. Although *MTND4P12* was significantly upregulated in AAs, in the present study, *AURKB* itself did not exhibit significant differential expression, suggesting that additional mechanisms may be involved in regulating its activity.

Pathway enrichment analyses provided further insights into the biological processes and signaling cascades dysregulated in a race- and MD-Cluster-related manner. The downregulation of DNA replication and damage response pathways in AAs and upregulation of histone genes *H2AC7*, *H2AC14*, *H2BC9*, *H2BC12*, *H2BC14*, and *H2BC15* in AAs is notable because downregulation of histone pathways (H2A and H2B) is associated with anthracycline sensitivity in breast cancers^54^. In addition, pathways involved in breast cancer regulation by stathmin1 are downregulated in AA patients. Stathmin-1 is a cytoplasmic phosphoprotein involved in mitotic spindle formation and cell division^55^. Its downregulation in AA TNBCs indicates a reduced microtubule formation, which could affect cell division, leading to a differential response to treatments targeting microtubule dynamics, such as taxane-based chemotherapies^56,57^.

Comparison of gene expression based on MD-Clusters revealed significant upregulation of genes involved in mitochondrial tRNA processing in MD-Cluster 1 (enriched for AA tumors), and genes related to triglyceride metabolism and docosahexaenoic acid (DHA) pathways in MD-Cluster 2 (predominantly EA tumors). The DHA pathway induces apoptosis in cancer cells by activating the MAPK signaling pathway and suppressing Akt phosphorylation^58,59^, and may potentially promote tumor growth, progression, and metastasis in breast cancers^60^. It is therefore possible that upregulation of DHA pathways in MD-Cluster 2 may induce apoptotic effects in EA patients, potentially underlying better disease-free survival.

We performed xCell-based cell enrichment analyses to identify the various cell types associated with race and MD-Clusters. We observed that Th1 cells, MEPs, and pro B-cells were enriched in AA patients; macrophage M2 cells were more abundant in EA patients, with preadipocytes enriched in MD-Cluster 2, dominated by CAs. At first glance, the immune cell findings appear to be at variance with previous studies suggesting the poorer outcomes in breast cancer in general among AA women may be linked in part to higher M2 abundance in AA breast tumors than EA tumors^61,62^. However, similar to our findings, other TNBC-focused studies^63^ have observed lower levels of M2 macrophages in AA women, and nuanced interpretation of our data underscores the complexity of these relationships. Our observation of a linear relationship between M1 and M2 abundance in AA but not in EA women is in keeping with the link between mixed M1/M2 phenotype and tumor aggressiveness^64^. Very interestingly, we also observed that the subset of AA women with the highest tumor M2 abundance had poorer DFS compared with EA women regardless of their tumor M2 abundance. This suggests that there is still a relationship between high M2 abundance and poor outcomes in AA women in spite of the overall lower M2 abundance in AA women compared with EA women.

Our integrated analysis of microbial and transcriptional clusters identified significant host-microbe associations that potentially influence DFS in patients. Cluster 26 emerged as particularly notable, with strong positive correlations between *SPDYE2B* and *Hafnia*. Both of these features were significantly abundant in AA patients. A high abundance of *Hafnia* and *SPDYE2B* was associated with worse DFS, suggesting that, for subsets of TNBC patients, these features may synergize in influencing tumor biology and patient outcomes.

The identification of race-specific host-microbe interactions in TNBC offers new insight into how tumor biology and health disparities may differ across populations. The strong positive correlation between *SPDYE2B* expression and *Hafnia* abundance in AA patients, coupled with their shared association with poor DFS, suggests a synergistic relationship that may contribute to the observed racial disparities in TNBC outcomes. This host-microbe axis potentially creates a feedback loop where microbial metabolites or virulence factors influence cell cycle regulation through the Speedy/Ringo pathway, while altered host gene expression may reciprocally modify the tumor microenvironment to favor specific microbial populations. The differential immune cell compositions observed—with AA tumors showing higher Th1 cell abundance and EA tumors exhibiting greater M2 macrophage infiltration—may serve as both drivers and consequences of these distinct microbial communities. Importantly, our observation that AA women with high M2 abundance experienced the poorest survival outcomes suggests that the interplay between immune phenotype and race may create unique vulnerabilities that could be targeted as therapeutic avenues. These findings highlight the necessity of incorporating both host genetics and microbial ecology into precision medicine approaches for TNBC, as traditional biomarkers may inadequately capture the complexity of tumor biology across diverse populations.

Our current study serves as an important proof-of-concept that demonstrates the feasibility of identifying race-related host-microbe interactions in TNBC. While our sample size is small, it is comparable to other exploratory microbiome studies on breast cancer tissues^65–67^. In addition, no two studies have produced the exact same microbial associations. This can be explained in part by the samples being collected from different geographical sites. We also hypothesize that the role of microbes in breast cancer is primarily facultative rather than causative and that the effects are subserved by microbial products and/or metabolites which may be more similar in function than bacterial identity and taxonomy may suggest. Bacteria identified in different studies will ultimately help to narrow down the culprit metabolites in future work.

We have presented a novel integrative analytical framework for identifying host-microbe associations that can be applied to larger cohorts to validate race-associated interactions and their clinical implications in TNBC. Larger cohort studies incorporating multi-omics data and comprehensive clinical annotations would facilitate the identification of robust microbiome-host signatures predictive of disease outcomes, paving the way for the development of novel prognostic biomarkers and therapeutic strategies tailored to distinct patient populations.

## Methods

### Host transcriptome analysis

The samples were collected as macro-dissected breast tumor tissues during routine surgical procedures at UAB between 2001 and 2012^17^. A retrospective cohort of formalin-fixed, paraffin-embedded (FFPE) archival tissues from the Division of Anatomic Pathology at UAB was subjected to RNA extraction using standard protocol. The extraction of RNA from the macro-dissected samples was subjected to RNA extraction using standard protocol. Raw sequencing reads were assessed for quality using FastQC v0.11.8 (https://github.com/s-andrews/FastQC). Adapters and low-quality bases were trimmed from the raw reads with Trimmomatic v0.36^68^ to retain only high-quality sequences for downstream analysis. The trimmed reads were aligned to the human reference genome (GRCh37) using the HISAT2 aligner v2.0.4^69^. Gene type annotations were performed with GENCODE version 46. Following read alignment and rRNA removal, the RNASeq data were assembled into transcript models using Stringtie v1.3.3^69^. Differential expression (DE) analysis between race groups was performed using the Bioconductor package DESeq2 version 1.36.0^70^ to compute log2FoldChange (L2FC) and Benjamini-Hochberg (BH)-based False Discovery Rate (FDR) adjusted p-values. Statistical significance was determined at an FDR threshold of 0.05.

### Microbial transcriptome analysis

To identify microbial sequences from tumor RNASeq data (n = 36), we used the PathSeq pipeline^71^^,72^. PathSeq first removes low-quality and low-complexity reads. Host reads are then filtered out through a two-step process. First, k-mer matching (default, *k* = 31) rapidly identifies short sequences of human origin. Second, the Burrows-Wheeler Aligner (BWA-MEM)^73^ iteratively aligns reads to the human reference genome and removes those with defined identity and coverage. Unaligned non-host reads are subsequently aligned to a microbial genome database (RefSeq: archaea, bacteria, fungi, protozoa, and virus) using BWA-MEM^73^. The mapped reads were then used to derive the microbial read counts at each taxonomic level in each sample.

Using the microbial data, we performed Spearman rank correlation-based hierarchical clustering of tumors with average linking to determine microbe-derived clusters (MD-Clusters). To identify microbes with robust race-specific abundance differences between AA and EA cohorts, we used the comparative marker selection tool in Morpheus (https://software.broadinstitute.org/morpheus/). Microbial transcripts count data were log_2_(counts+1) transformed and analyzed by t-tests between AA and EA groups. BH-based false discovery rate (FDR) adjustment was applied to select significant markers at an FDR threshold of 0.05.

### Differential expression of host genes based on MD-Cluster profiles

Similar to the race differential expression analysis, MD-Cluster-based differential expression was performed using DESeq2 version 1.36.0^67^. Log_2_(fold changes) and FDR-adjusted p-values were computed to identify genes with significant expression differences between MD-Cluster groups. An FDR threshold of 0.05 was used to determine statistical significance.

### Pathway analysis

Pathway analysis was conducted using the IPA software (QIAGEN) by using the gene expression data derived from both race and MD-Cluster stratifications. Coding genes with an absolute log_2_ fold change ( | L_2_(FC)|) ≥ 1 and a p < 0.05 were subjected to pathway analysis and construction of a protein-protein interaction (PPI) network. Pathways or networks with absolute Z-scores ≥ 2 and p < 0.05 were considered significantly enriched.

### Integration of microbiome and transcriptomic data

To identify associations between the microbiome composition and host gene expression, microbial transcript abundance and gene expression data were combined into a single matrix. This matrix was subjected to hierarchical clustering using Spearman correlation with the average linkage method to generate 1000 clusters. Of these, clusters with only bacteria or only host genes (homologous clusters) were filtered out, but those containing at least one bacterium and at least one host gene (heterologous clusters), with data representation (non-zero) in ≥50% of samples, were retained. Subsequently, high-confidence heterologous clusters were identified using a cutoff of Spearman correlation >0.7 between host genes and bacteria (Supplementary Fig. 5).

### Cell-type enrichment analysis

To investigate cellular heterogeneity in TNBC tumors, we performed gene-signature-based, cell-type enrichment analysis using xCell version 1.1.0 (https://github.com/dviraran/xCell/tree/master). This analysis generated xCell scores for various cell types. Further, we assessed the association of these xCell scores with race and MD-Cluster by using the Wilcoxon rank-sum test.

### Disease-free survival

Kaplan-Meier (KM) curves were generated to determine disease-free survival (DFS) using ‘survival’, ‘survminer’, and ‘ggplot2’ R packages. Curves were stratified by race, MD-Cluster, treatment types, or microbe/gene-based abundance (high/low groups split by median). Survival distributions were compared using the log-rank test, with p < 0.05 considered statistically significant. The Cox proportional hazards model was used to analyze the impact of *Hafnia* and *SPDYE2B* abundance on DFS, adjusting for race, MD-Cluster, stage, and treatment type.

## Supplementary information

Supplementary information

Supplementary data1–8

## Data Availability

The datasets are available in the BioProject database under Accession No. PRJNA598161. All analyses were performed using publicly available software and standard protocols. No custom code was developed for this study.

## Code availability

The specific software versions and parameters used are detailed in the Methods section.
