# Supplementary material for: Race-related host and microbe transcriptomic signatures in triple-negative breast cancer
Source: NPJ Breast Cancer. 2025 Aug 8;11:87. doi: 10.1038/s41523-025-00806-y (PMC12334597; doi:10.1038/s41523-025-00806-y)
Supplement: Supplementary file 1 — Supplementary information [file 41523_2025_806_MOESM1_ESM.pdf]

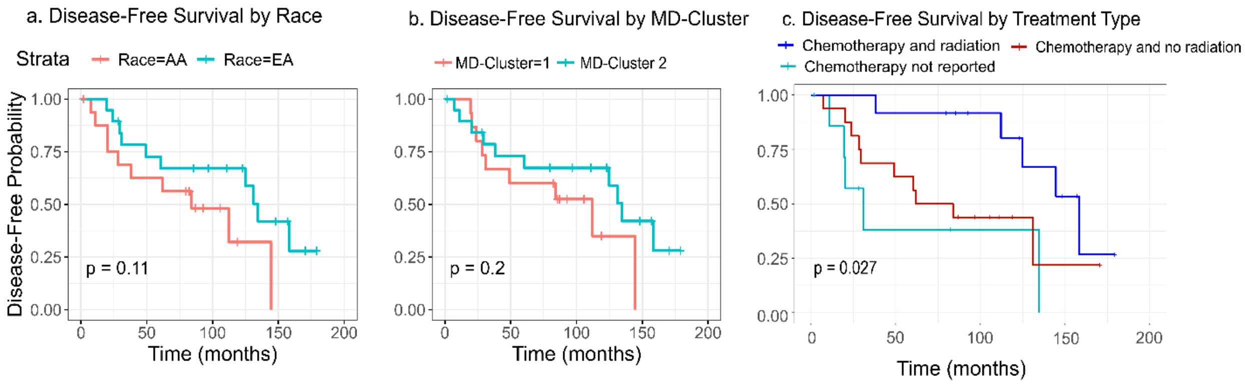

Supplementary Figure 1: Disease-Free Survival Analysis. Kaplan-Meier curves illustrating disease-free survival (DFS) stratified by: a. Race, b. MD-Cluster, c. Treatment type in TNBC patients. Statistical analysis revealed no significant differences in DFS based on race or MD-Cluster. However, significant variations in DFS were observed among different treatment types.

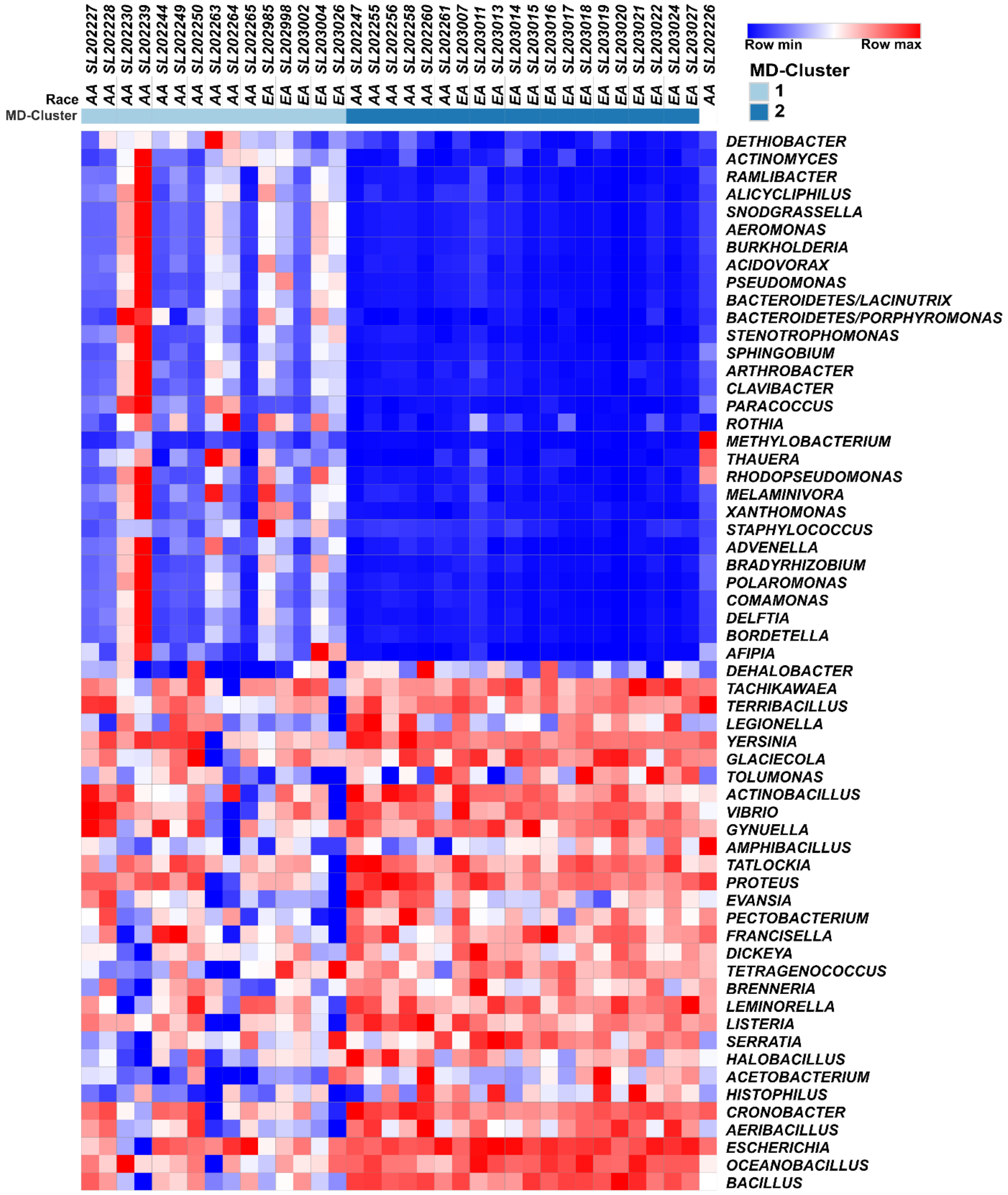

Supplementary Figure 2: Differential Abundance of Tumor Microbiome in TNBC Patients by MD-Cluster. Comparative marker selection analysis was performed using t-test (FDR < 0.05) to identify taxa with differential abundance between MD-Cluster 1 and 2 patients.

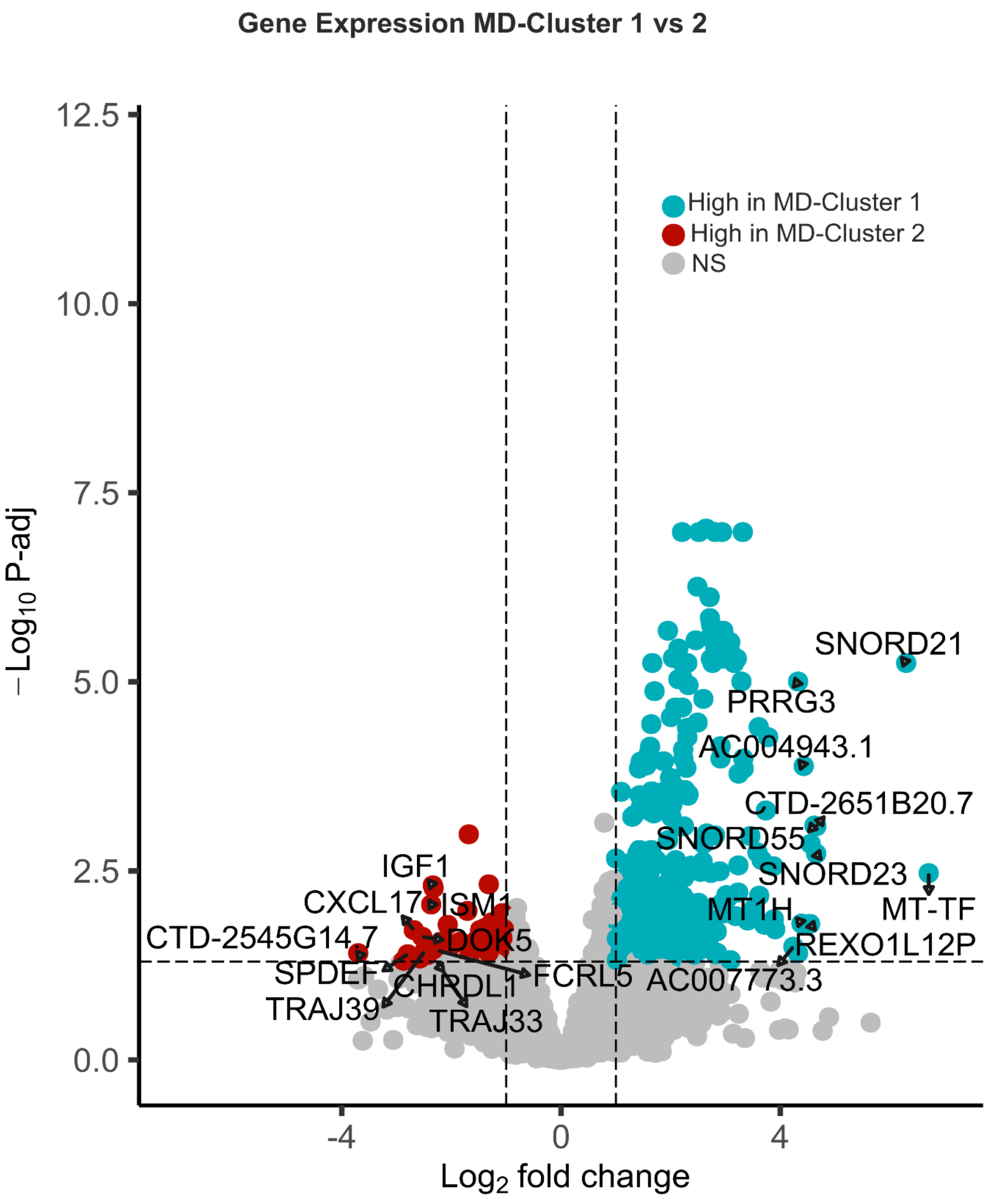
Gene Expression MD-Cluster 1 vs 2
High in MD-Cluster 1
High in MD-Cluster 2
NS
$-Log_{10}$ P-adj
Log_2 fold change
12.5
10.0
7.5
5.0
2.5
0.0
-4
0
4
SNORD21
PRRG3
AC004943.1
CTD-2651B20.7
SNORD55
SNORD23
MT-TF
MT1H
REXO1L12P
AC007773.3
FCRL5
IGF1
CXCL17
ISM1
DOK5
CTD-2545G14.7
SPDEF
CHRDL1
TRAJ39
TRAJ33

Supplementary Figure 3: **Differential gene expression between MD-clusters 1 and 2.** Volcano plot showing the log2 fold change and adjusted p-values for all differentially expressed genes between MD-Cluster 1 and 2 groups (|log2FoldChange| ≥ 1, padj < 0.05).

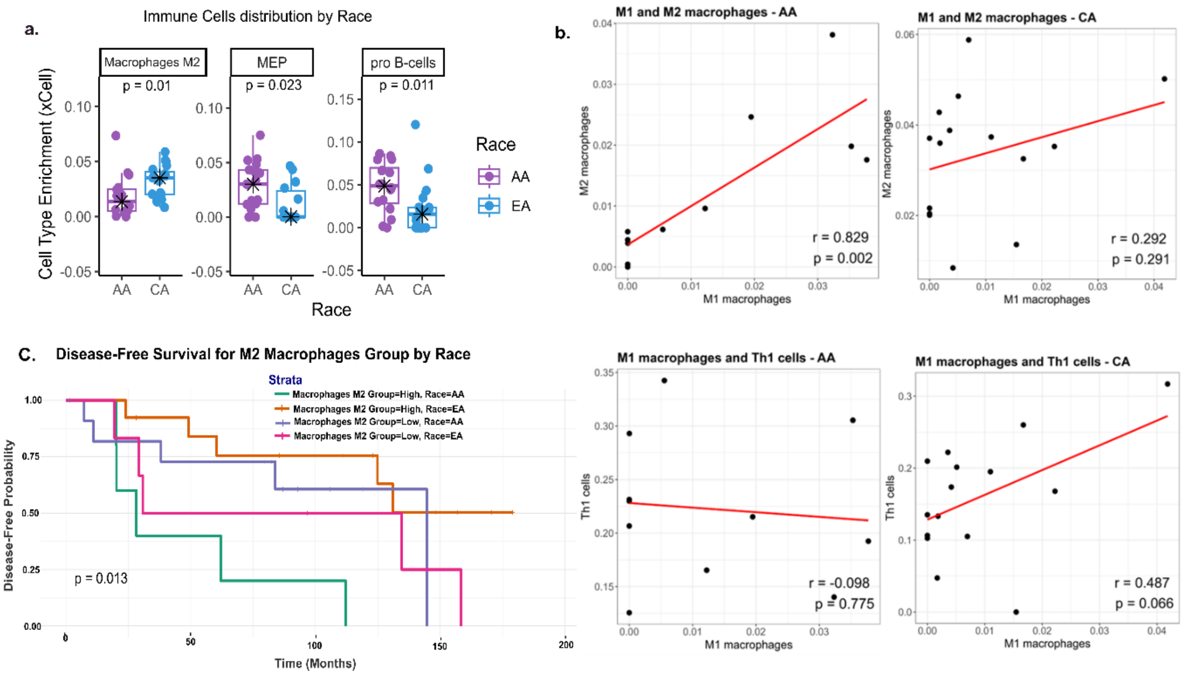

Supplementary Figure 4: **Immune cell distribution, correlation, and survival analysis by race.** a. Boxplots showing the enrichment of M2 macrophages, megakaryocyte-erythroid progenitors (MEP), and pro B-cells across AA and EA tumors, based on xCell analysis. Statistical significance between groups is indicated by the p-values. b. Scatterplots illustrating the correlations between M1 and M2 macrophages, as well as M1 macrophages and Th1 cells, stratified by race (AA and EA). Pearson correlation coefficients (r) and p-values are shown for each correlation. c. Kaplan-Meier curves for disease-free survival stratified by high and low M2 macrophage levels (demarcated by median vales) within AA and EA groups.

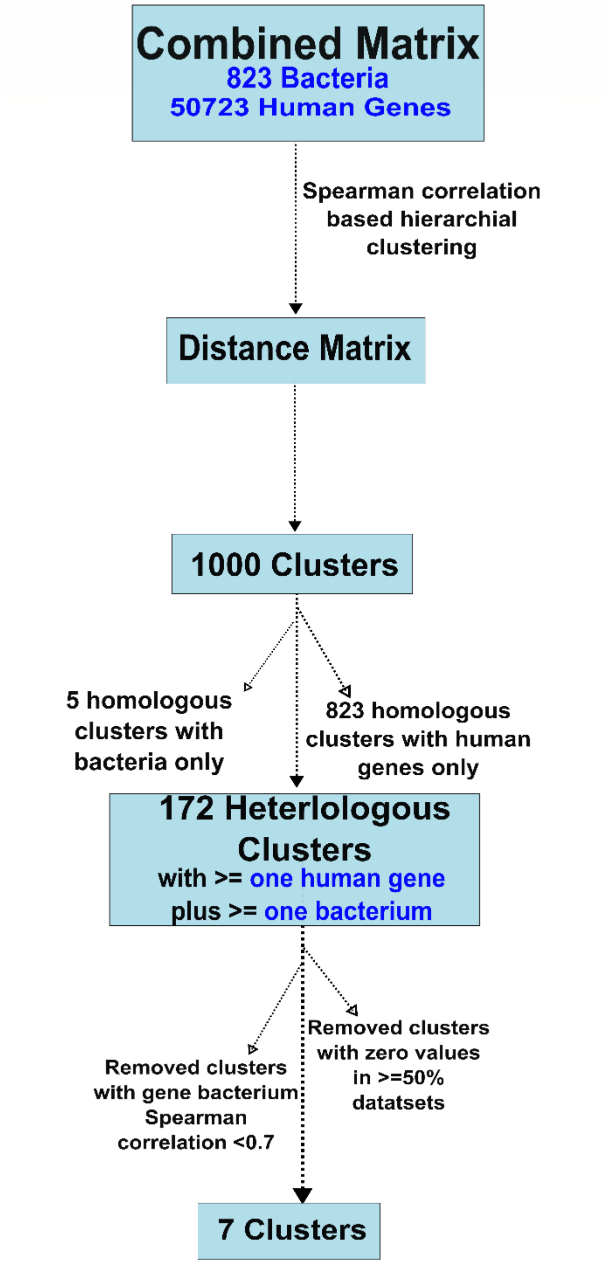

Supplementary Figure 5: Hierarchical clustering-based relationships between host-genes and bacteria. The diagram illustrates the method of integrating microbiome and transcriptomic data to identify selected correlated heterologous (gene-bacteria) clusters.

Supplementary Data 1: The abundance of the microbial community in the TNBC cohort (n=36) determined using PathSeq. Microbial sequences were extracted from tumor RNA-Seq data using the PathSeq computational pipeline. The workflow begins with quality control, removing low-quality reads. Human-derived sequences were eliminated through a dual-step filtration: initial k-mer matching (k=31) for rapid human sequence identification, followed by iterative BWA-MEM alignment against the human reference genome to remove reads meeting specific identity and coverage criteria. Remaining non-host reads undergo alignment to a comprehensive microbial reference database (RefSeq: archaea, bacteria, fungi, protozoa, and viruses) via BWA-MEM, generating taxonomic-level microbial read counts for each sample.

Supplementary Data 2: The DESeq2 results, with log2FC, p-value, and p-adj values between AA and CA cohorts. Comparative transcriptomic analysis was performed between racial groups using DESeq2 v1.36.0. The analysis quantified log2FoldChange values and applied Benjamini-Hochberg correction for multiple testing, generating False Discovery Rate-adjusted p-values. Genes demonstrating statistically significant differential expression were identified using an FDR cutoff of 0.05.

Supplementary Data 3: Enriched Canonical Pathways with absolute Z-score ≥ 2 and p-value < 0.05 in TNBC cohort based on race. Functional pathway enrichment analysis was performed using QIAGEN's Ingenuity Pathway Analysis platform, integrating race-stratified gene expression profiles. Input criteria included protein-coding genes exhibiting absolute L2FC ≥1 and raw p-values <0.05. Significantly enriched pathways and protein-protein interaction networks were defined by absolute Z-scores ≥2 combined with p-values <0.05.

Supplementary Data 4: The DESeq2 results, with log2FC, p-value, and p-adj values between MD-Cluster 1 vs 2 cohorts. Transcriptome-wide comparison between MD-defined clusters was performed using DESeq2 v1.36.0. L2FC calculations and Benjamini-Hochberg multiple testing correction generated FDR-adjusted significance values, with differential expression determined at FDR threshold of 0.05.

Supplementary Data 5: Enriched Canconical Pathways with absolute Z-score ≥ 2 and p-value < 0.05 in TNBC cohort based on MD-Cluster. Pathway analysis was conducted using the Ingenuity Pathway Analysis (IPA) software (QIAGEN) by using the gene expression data derived from MD-Cluster stratification. Coding genes with an absolute log2 fold change (|L2(FC)|) ≥ 1 and a p-value < 0.05 were subjected to pathway analysis and construction of a protein-protein interaction (PPI) network. Pathways or networks with absolute Z-scores ≥ 2 and p-values < 0.05 were considered significantly enriched.

Supplementary Data 6: The hierarchical clustering using Spearman Rank correlation method was performed for integrated analysis of microbial and host genes to identify correlated gene-bacteria clusters. To identify associations between the microbiome composition and host gene expression, microbial transcript abundance and gene expression data were combined into a single matrix. This matrix

was subjected to hierarchical clustering using Spearman correlation with the average linkage method to generate 1000 clusters. Homologous clusters (containing exclusively bacteria or host genes) were excluded, while heterologous clusters (containing both bacterial and host components) with ≥50% sample representation were retained. High-confidence associations were identified using Spearman correlation coefficients >0.7 between host genes and bacterial species.

Supplementary Data 7: Highly correlated host gene and microbial pairs-associated microbes and their correlation values. Curated collection of strongly correlated host gene-microbial species associations with their respective correlation coefficients. This dataset encompasses heterologous clusters meeting dual criteria: mixed bacterial-host gene composition with ≥50% sample coverage and Spearman correlation values exceeding 0.7 between host transcripts and microbial abundances.

Supplementary Data 8: Cox proportional hazards model results showing variables and their impact on hazard ratio. Multivariable survival analysis was performed examining the prognostic impact of *Hafnia* abundance and *SPDYE2B* expression on disease-free survival outcomes. The Cox regression model incorporated adjustment variables including race, MD-Cluster assignment, tumor stage, and treatment modality to assess independent hazard ratios.
